# Optimization of Extraction Conditions of Areca Seed Polyphenols and Evaluation of Their Antioxidant Activities

**DOI:** 10.3390/molecules191016416

**Published:** 2014-10-13

**Authors:** Wei-Min Zhang, Wu-Yang Huang, Wen-Xue Chen, Lin Han, Hai-De Zhang

**Affiliations:** 1 College of Food Science, Hainan University, Haikou 570228, China; E-Mails: zhwm1979@163.com (W.-M.Z.); hnchwx@163.com (W.-X.C.); hanlin730@163.com (L.H.); 2 Jiangsu Academy of Agricultural Sciences, Nanjing 210014, China

**Keywords:** antioxidant activities, *Areca catechu* L., extraction, polyphenols

## Abstract

Polyphenols are functional compounds in plants, which possess many bioactivities beneficial for humans. The aim of this study was to establish a highly efficient method for extracting polyphenol compounds from areca seeds and further to identify polyphenols and antioxidant properties of the seeds. A quadratic general rotary unitized design was used to determine the optimal extraction process. The polyphenols were identified using LC-TOF-MS. By comparison with ascorbic acid (V_c_), the antioxidant activities of the ethanol extracts were evaluated using three complementary *in vitro* assays: inhibition of the DPPH (1,1-diphenyl-2-picrylhydrazyl) radical-scavenging activity, hydroxyl radical-scavenging activity, and reducing ability. The two major polyphenols obtained were epicatechin and syringic acid. The ethanol extracts of areca seeds showed significantly greater antioxidant activity (*p* < 0.05) than V_c_ using the DPPH and reducing power assay, but lower ability (*p* < 0.05) using the hydroxyl radical assay. The results indicate that the areca seed is an excellent food material with potential antioxidant properties.

## 1. Introduction

The areca nut (*Areca catechu* L. from the Palmaceae family) is a tropical fruit, which is also called betel nut and is widely distributed in different parts of the world. As one of the major tropical crops, more than 223,800 metric tons of areca fruit were produced in Hainan province of China in 2013. About 90% of the areca harvest is available as commercial preparations which are produced on a large scale [1]. The processing involves husking fruits, removing embryos, and drying nuts in the sun or with artificial heat or sometimes smoking. The dried product is graded according to harvest, color, shape, and size. Nuts may first be boiled to reduce tannin content of nuts, and then dried [2]. The seeds of areca nut account for 45% of the total weight of fruit. However, they are discarded during the process of producing the betel quid chewing (BQC) and/or areca nut chewing (ANC). In Hainan and Hunan provinces, the annual yield of areca seeds is above 100,710 metric tons, which leads to much wastage and a serious environmental problem. Therefore, a new commercial industry for the areca nut to reutilize the waste is emerging. For more economical and efficient utilization, the characteristics and composition of the commercial product needs to be scientifically studied. The areca nut contains many nutritional and functional components with different bioactivities. Since the 4th century A.D., humans have consumed the areca nut as food and medicine. Now, it is estimated that over 600 million individuals consume areca nut world-wide [2]. In India, areca nut has been described as a therapeutic agent in old scripts, such as Vagbhata (4th century) and Bhavamista (13th century). It was recommended for the treatment of many diseases, such as leucoderma, leprosy, anaemia, and obesity. In China, it has been used as a vermifuge to eliminate intestinal worms since the 6th century and is still employed in some areas [3]. In Philippines, the flowers are sometimes added to salads. Various medicinal preparations use the nuts, husks, young shoots, buds, leaves, or roots [2]. These pharmacology activities are attributed to abundant phenolic compounds in the areca nuts.

The phenolic compounds or polyphenols, secondary metabolites of plants, are phytochemicals that exhibit antioxidant activity and consequently possess a beneficial physiological effect [4,5]. Polyphenols are the most prevalent antioxidant phytochemicals in the plant kingdom and reportedly possess both singlet oxygen quenching activity and radical scavenging activity [6]. They are able to delay lipid oxidation in foodstuffs and biological membranes, and they can act as a prophylactic agent, which have motivated research into food science and biomedicine [7]. Considering their bioactivity and wide distribution, these substances are potential natural antioxidants, and the vegetable source can be considered as functional food [8]. Although available studies have already demonstrated that areca fruit contains many phenolics and tannins [9], little is known about the antioxidant activities of polyphenols in areca seed.

Different conventional liquid solvent extraction strategies have been employed for the extraction of phenolics from plants like Soxhlet extraction, maceration, microwave-assisted extraction, ultrasound-assisted extraction, high hydrostatic pressure extraction and supercritical fluid extraction with CO_2_ *etc*. [10,11]. The extraction rate may be improved by choosing the best combination of process variables, such as solvent composition, extraction temperature, liquid to solid ratio and extraction time [12]. The extraction process has to be optimized depending upon the nature of the sample and purpose of the study [13]. In this study, we endeavored to develop an efficient and effective extraction method of polyphenols for areca seeds. In addition, the extracted polyphenols were profiled, and the antioxidant properties of different areca extracts were evaluated using several biochemical assays, including DPPH (1,1-diphenyl-2-picrylhydrazyl) radical-scavenging activity, hydroxyl radical-scavenging activity, and reducing power. To the best of our knowledge, this is the first report on optimization of different extraction conditions of phenolic antioxidants from areca seeds.

## 2. Results and Discussion

### 2.1. Optimized Extraction Conditions of Areca Polyphenols

The quadratic regression rotational combination design incorporating the three factors was used to optimize the extraction process, and the yield of polyphenols from areca seed was designated to be the evaluation index. In order to determine the optimal extraction process, a mathematical regression model was analyzed by frequency analysis as shown in Table 1. The equation used was:

*Y* = 16.10445 + 1.46743 × *X*_1_ − 1.88977 × *X*_2_ + 0.10339 × *X*_3_ − 1.44227 × *X*_1_^2^ − 1.49885 × *X*_2_^2^ − 1.67921 × *X*_3_ × *X*_3_ + 0.91125 × *X*_1_ × *X*_2_ + 0.09625 × *X*_1_ × *X*_3_

**Table 1:** The test scheme and its results in quadratic general rotary unitized design of areca seed.

No	*X*_0_	*X*_1_	*X*_2_	*X*_3_	*X*_1_*X*_2_	*X*_1_*X*_3_	*X*_2_*X*_3_	*X*_1_^2^	*X*_2_^2^	*X*_3_^2^	*Y* (%)
1	1	1	1	1	1	1	1	1	1	1	12.12
2	1	1	1	−1	1	−1	−1	1	1	1	12.67
3	1	1	−1	1	−1	1	−1	1	1	1	15.28
4	1	1	−1	−1	−1	−1	1	1	1	1	14.11
5	1	−1	1	1	−1	−1	1	1	1	1	7.05
6	1	−1	1	−1	−1	1	−1	1	1	1	6.48
7	1	−1	−1	1	1	−1	−1	1	1	1	12.35
8	1	−1	−1	−1	1	1	1	1	1	1	13.07
9	1	−1.682	0	0	0	0	0	2.828	0	0	10.37
10	1	1.682	0	0	0	0	0	2.828	0	0	13.23
11	1	0	−1.682	0	0	0	0	0	2.828	0	14.41
12	1	0	1.682	0	0	0	0	0	2.828	0	8.87
13	1	0	0	−1.682	0	0	0	0	0	2.828	10.85
14	1	0	0	1.682	0	0	0	0	0	2.828	11.41
15	1	0	0	0	0	0	0	0	0	0	16.08
16	1	0	0	0	0	0	0	0	0	0	16.16
17	1	0	0	0	0	0	0	0	0	0	16.00
18	1	0	0	0	0	0	0	0	0	0	16.24
19	1	0	0	0	0	0	0	0	0	0	16.32
20	1	0	0	0	0	0	0	0	0	0	15.92

After optimizing the combination of different factors by computer, the optimum extraction parameters were: temperature of 69.15–77.19 °C, time of 115.70–125.87 min, and liquid to solid ratio of 10.13–10.92. The optimum extraction conditions were determined as 70% ethanol, liquid to solid ratio of 11:1 (*v*/*w*), temperature 70 °C, and time 120 min, which led to a yield of polyphenols up to 16.37% in the actual experiment. This was not significantly different to the theoretical value of 16.48% achieved by calculation with the equation (*p* > 0.05).

Han *et al*. reported the optimal extracting conditions of total phenol from betel nut seed as 70% ethanol, extraction temperature 58 °C, extraction time 4 h, and liquid to solid ratio 47 mL/g. The predicted value and measured value of total phenol were 148.09 mg/g and 146.63 mg/g, respectively [14]. Using ultrasonic wave to extract total phenol in the areca nut seed, the optimal extraction process were 58 °C, 42 min, and liquid to solid ratio 53 mL/g with the predicted and measured value of total phenol at 164.74 mg/g and 160.95 mg/g, respectively [15]. Our optimization of the extraction got the extraction rate of 16.37% (*i.e*., 163.7 mg/g), which was similar to Han’s reports.

### 2.2. Preliminary Identification of Polyphenols in Areca Seed

**Figure 1:**
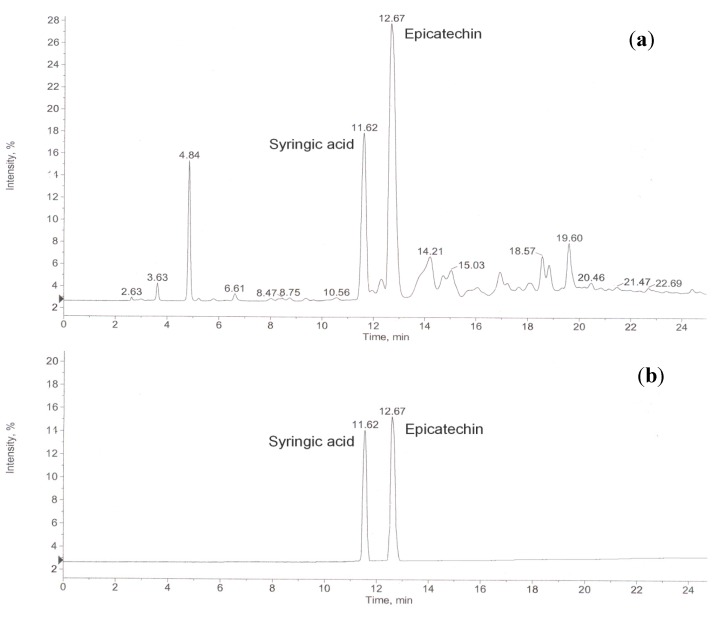
Typical HPLC-UV chromatograms of (**a**) 70% ethanol extracts of areca seeds and (**b**) mixed standard solution of syringic acid and epicatechin.

**Figure 2:**
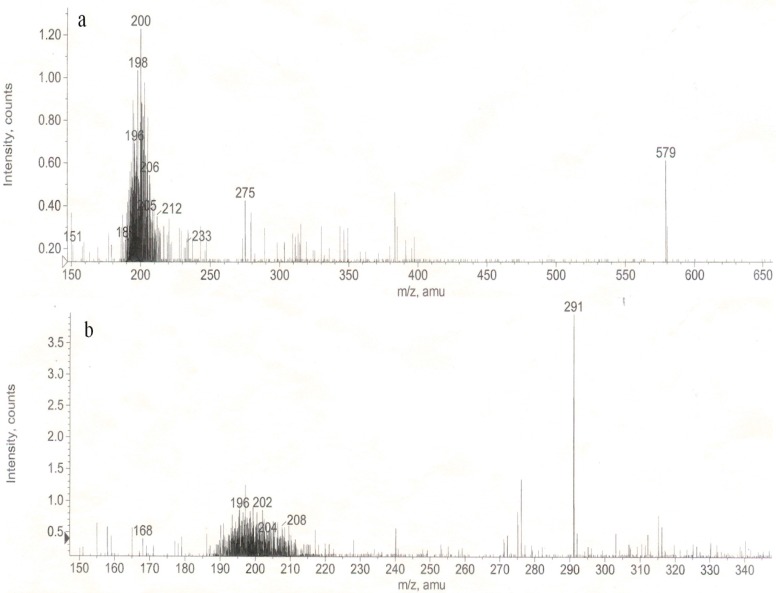
CID MS–MS spectra of (**a**) mass spectrum of syringic acid and (**b**) mass spectrum of epicatechin.

**Figure 3:**
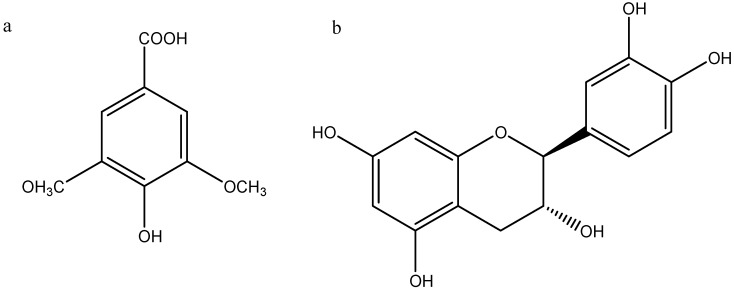
Structures of (**a**) syringic acid and (**b**) epicatechin.

Recently, HPLC–MS and GC–MS have been applied to unambiguously identify the structures of flavonoids and phenols in plant extracts and biological samples with great success [16,17]. In addition, HPLC–MS has been successfully employed for the identification of phenolic compounds in food samples [18]. In the present study, suspected polyphenols were profiled by HPLC chromatograms (Figure 1). Elemental composition calculations from exact mass measurements (typically within 5 ppm) were used to make assignments for unknown compounds. Only two components in areca seed have been identified in this preliminary study. The two main chromatographic peaks were attributed by MS (Figure 2), with epicatechin being the most abundant compound in the areca seed, and the other major compound was syringic acid. The compounds identified are shown in Table 2. The structures of these two compounds are showed in Figure 3.

**Table 2:** Spectral information of LC-TOF-MS for two phenolic compounds.

Peak No.	Phenolic Compounds	MW	[M-H]^+^ (Frag.MS^2^*m*/z)	Retention Time (min)
1	Syringic acid	198	199 (183,154)	11.62
2	Epicatechin	290	291 (246,180,126)	12.67

*Areca catechu* L contains diverse phenolics composition, including condensed tannins, hydrolyzed tannins, lignans, stilbenes, flavane, flavonoids (mainly catechin and epicatechin), anthocyanin, and simple phenolic acids [4,19,20,21,22]. Wang *et al*. found catechin was the highest content in *Areca catechu*, followed by tannic acid, epigallocatechin, epicatechin, epigallocatechin gallate, and gallic acid [23]. Many phenolic constituents of areca fruits have been reported, such as flavonoids (e.g., luteolin, isorhamnetin, chrysoeriol, jacareubin, mignonette, and (±)-4',5-dihydroxy-3',5',7-trimethoxyflavonone), tannin (e.g., Arecatannin A1, Arecatannin A2, Arecatannin A3, Arecatannin B1, Arecatannin B2 and Arecatannin C1), phenolic acids (e.g., p-hydroxybenzoic acid and hydroxycinnamic acid), anthocyanins (e.g., colorless pelargonidin dimers), tea polyphenols dimers, catechin, and lignin [24,25,26]. The total phenolic content of areca seeds was higher than that of the fruits [27], however, few reports that detail the polyphenols in areca seeds could be found. In this study, epicatechin and syringic acid were detected as the two major phenolic compounds. Zhang *et al*. and Pu also identified epicatechin in areca inflorescence and nut as the major polyphenol using RP-HPLC-PAD and HPLC-MS, respectively [28,29].

### 2.3. Antioxidant Activities of Areca Seed

Available studies of areca nuts concern the properties of phenolic compounds in fruits [30,31], however, few reports relate to the nutritional compositions and antioxidants of areca seeds. Areca seeds, the residue of areca fruits, are normally discarded during BQC processing, which were found to contain much higher antioxidant activity than the areca husk by using several methods to assess antioxidant activity [27].

The additive and synergistic effects of phytochemicals are responsible for their potent bioactive properties and the benefit of a diet rich in fruits and vegetables is attributed to the complex mixture of phytochemicals [32]. This explains why no single antioxidant can replace the combination of natural phytochemicals to achieve maximum health benefits. Therefore, we carried out the assays using whole extracts instead of individual compounds. In this study, several biochemical assays were used to screen the antioxidant properties of the areca seed extract: DPPH radical-scavenging activity, hydroxyl radical-scavenging activity and reducing power. Individual assays were carried out on each extract separately.

Analysis of results reveals that the antioxidant activity of areca seed had a linear correlation with the extract concentration (DPPH radical scavenging: *Y* = 6.576*x* + 58.99, *R*^2^ = 0.9159; Hydroxyl radical scavenging activity: * Y* = 13.202*x* + 2.805, * R*^2^ = 9928; Reducing power: *Y* = 0.1742*x* + 1.2279, * R*^2^ = 0.9890). High activity was recorded even at low extract concentrations. As shown in Table 3, the DPPH and reducing power assays indicated that the antioxidant activity of areca seed extracts (EC_50_ was 0.409 and 0.188 mg/mL, respectively) was significantly greater (*p* < 0.05) than that of ascorbic acid (V_c_), however, the hydroxyl radical assay showed it (EC_50_ was 3.75 mg/mL) to be significantly lower than V_c_ (*p* < 0.05). Han *et al*. also found the betel nut seed extract presented strong antioxidant activities to DPPH and ABTS radical with EC_50_ at 145.62 μg/mL and 139.35 μg /mL, respectively [14]. It was interesting to note that areca seed extracts showed greater efficiency in scavenging DPPH radicals and reducing power than in scavenging hydroxyl radicals. It might indicate that the areca seed possessed antioxidant capacity mainly because it could scavenge DPPH radical and could convert Fe^3+^/ferricyanide complex to the ferrous form.

**Table 3:** Correlations established between the concentration of extracts with antioxidant activity EC_50_ values.

EC_50_ (mg/mL)	DPPH Radical Scavenging	Hydroxyl Radical Scavenging Activity	Reducing Power
Areca seed	0.409 ^b^	3.575 ^a^	0.188 ^b^
Ascorbic acid	0.964 ^a^	0.557 ^b^	0.401 ^a^

Data followed by different letters in the same column are significantly different at 0.05 probability level (df = 3).

## 3. Experimental Section

### 3.1. Plant Materials and Sample Preparation

Areca seed was obtained from the areca (*Areca catechu* L.) plant grown in Hainan, China. The areca fruit was harvested in June 2008. The samples were cleaned, washed with distilled water, cut into small pieces, dried overnight in an air dryer at 40 °C, and ground to particles by a 20-mesh sieve.

### 3.2. Chemicals and Reagents

DPPH (1,1-diphenyl-2-picrylhydrazyl) and catechin were purchased from Sigma Chemical Company (St. Louis, MO, USA). Vc (ascorbic acid) was obtained from Guangzhou Chemical Reagent Factory, Guangzhou, China. All other reagents were of analytical grade.

### 3.3. Quadratic General Rotary Unitized Design

Ethanol is a safe and effective organic solvent to extract polyphenols from plants. Our previous study showed that 70% ethanol was found to be the optimum concentration for the extraction solvent. The effects of time (*X*_3_), temperature (*X*_2_), and liquid to solid ratio (*v*/*w*) (*X*_1_) on the yield of polyphenols were evaluated, and the yield of polyphenol (*Y*) from areca seed was designated as the evaluation index. The quadratic regression rotational combination design which incorporated the three factors (time, temperature, and liquid to solid ratio) was used to optimize the extraction process for polyphenols (*Y*) from areca seed. Determination of the base levels of polyphenols and the intervals during which their concentrations changed is shown in Table 4. The finely powdered and dried areca seed sample was extracted using 70% ethanol with different liquid to solid ratios (8, 8.8, 10, 11.2, 12) at different temperatures (65, 69, 75, 81, 85) for different times (90, 102, 120, 138, 150) by reflux. All experiments were conducted in triplicate.

**Table 4:** Experimental coding for factor and level.

Factor and Level	*X*_1_/Liquid to Solid Ratio (*v*/*w*)	*X*_2_/Temperature (°C)	*X*_3_/Time (min)
+1.682	12	85	150
+1	11.2	81	138
0	10	75	120
−1	8.8	69	102
−1.682	8	65	90
Δ_j_	1.2	6	18

### 3.4. Extraction of Polyphenols from Areca Seed

To extract the antioxidant compounds, the optimum extraction conditions were used. 5 g of the finely powdered and dried areca seed sample was extracted using 55 mL of 70% ethanol at 70 °C for 120 min by reflux. The extracts were filtered through Whatman No. 4 paper under reduced pressure, and then lyophilized by LGZ-10D Freezer Dryer (Sihuan Co., Beijing, China). All the samples were redissolved in 70% ethanol at a concentration of 5.0 mg/mL and analyzed for their content of polyphenols and antioxidant assay, including DPPH radical-scavenging activity, inhibition of hydroxyl radical-scavenging activity, and reducing power.

### 3.5. Determination of Polyphenol Content

The total phenolic content of the extracts was estimated using a colorimetric assay based on procedures described by Singleton and Rossi with some modifications [33]. Basically, 1 mL of sample was mixed with 1 mL of Folin and Ciocalteu’s phenol reagent. After 3 min, 1 mL of saturated sodium carbonate solution was added to the mixture and the volume was adjusted to 10 mL with distilled water. The reaction was kept in dark for 90 min. The absorbance was then read at 725 nm (UV-2450 Spectrophotometer, Shimadzu Co. Ltd., Kyoto, Japan). Catechin was used to produce a standard curve (2.0–12.0 µg/mL; *y* = 0.0631*x* − 0.0611; *R*^2^ = 0.9992).The results were expressed as mg of catechin equivalents/g of extract. The experiment was conducted in triplicate and the mean value was used.

### 3.6. Chromatographic Equipment

For the identification of polyphenols, HPLC-UV-MS analyses were carried out using a 1100 Series LC/MSD Trap-QTOF (Agilent Technologies, Palo Alto, CA, USA) equipped with an electrospray interface (ESI). The ion trap, working in the negative ionisation mode, was connected to an Agilent 1100 Series HPLC instrument consisting of an autosampler, solvent membrane degasser, binary pump, column thermostat, and a UV–Vis photodiode array detector. The HPLC 2D ChemStation Software with a ChemStation Spectral SW module was used for process guidance.

### 3.7. Analysis of Polyphenols by LC-TOF-MS

LC-TOF-MS analysis was conducted on an Aglient 1200–6100 system (Agilent Technologies, Santa Clara, CA, USA). The HPLC analytical separation was carried out using an Agilent Technologies Zorbax SB C_18_ column (250 mm × 4.6 mm, 5 µm) and a stepwise gradient mode of acetonitrile (solvent A) and 0.5% formic acid (solvent B) at a flow rate of 1.0 mL/min at 40 °C. A gradient elution used was 0–5 min, 92% B; 5–23 min, 92%–75% B. The scan range of ESI-MS was * m*/*z* 150–1200. Two fragmented ions were determined and the target mass was 400. Maximal accumulation time was 100 ms with 100% compound stability. A drying gas of 10 L/min was applied for ionization using nitrogen from generator and the nebuliser pressure was 15 psi. The spray voltage was +5500 V. The UV–Visible detector was set at 280 nm.

### 3.8. DPPH Radical-Scavenging Activity Assay

To evaluate the free radical-scavenging activity, the extracts were allowed to react with a stable free radical, 1,1-diphenyl-2-picrylhydrazyl radical (DPPH) [34]. Various concentrations (20, 40, 60, 80, 120, and 200 µg/mL) of areca extracts (0.3 mL) were mixed with 2.7 mL of 70% ethanol solution containing DPPH radicals (40 µg/mL). The mixture was shaken vigorously and left to stand for 60 min in dark (until stable absorbance values were obtained). The reduction of the DPPH radical was determined by reading the absorbance at 517 nm. The radical-scavenging activity (RSA) was calculated as a percentage of the DPPH discoloration, using the equation:

% RSA = [(*A*_DPPH_ − *A*_S_)/*A*_DPPH_] × 100

where *A*_S_ is the absorbance of the solution when the sample extract is added, and *A*_DPPH_ is the absorbance of the DPPH˙ solution [35]. The extract concentration showing 50% radical-scavenging activity (EC_50_) was calculated from the graph of the RSA percentage against extract concentration. Ascorbic acid samples were used as positive control.

### 3.9. Hydroxyl Radical-Scavenging Activity Assay

The hydroxyl radical-scavenging activity was evaluated using the method of Halliwell *et al*. [36] with a UV-visible spectrophotometer at a wavelength of 510 nm. The radical scavenging activity was calculated using the following equation:

Hydroxyl radical scavenging activity (%) = (*A*_0_ − *A*_i_)/*A*_0_ × 100

where *A*_0_ is the absorbance of the blank control at 510 nm, and *A*_i_ is the absorbance of the sample at 510 nm. Ascorbic acid was used as positive control and distilled water was used as blank control.

### 3.10. Reducing Power Assay

The reducing power of the extracts was assessed by the method of Oyaizu [37]. Various concentrations (20, 40, 60, 80, 120, and 200 µg/mL) of the extracts (2.5 mL) were mixed with 2.5 mL of 200 mM sodium phosphate buffer (pH 6.6) and 2.5 mL of 1% potassium ferricyanide. The mixture was incubated at 50 °C for 30 min. Following the addition of 2.5 mL 10% trichloroacetic acid (*w*/*v*), the mixture was centrifuged at 1000 rpm for 10 min. The upper layer (5 mL) was mixed with 5 mL deionised water and 1 mL 0.1% ferric chloride. The absorbance was measured spectrophotometrically at 700 nm [35]. A blank control was prepared using distilled water instead of extract. The values are presented as the means of triplicate analyses. The extract concentration providing an absorbance of 0.5 (EC_50_) was calculated from the graph of absorbance at 700 nm against extract concentration. Ascorbic acid samples were used as positive control.

### 3.11. Statistical Analysis

All experiments were conducted in triplicate, and statistical analyses were carried out according to the software DPS3.01 User’s Guides. The data were presented as mean ± SD (standard deviation). Determination of the significant differences of the means between various treatments of the areca seed was achieved using the T-test. Differences were considered significant with *p* value < 0.05.

## 4. Conclusions

In the quadratic general rotary unitized design for extraction of polyphenols from areca seed, the optimum conditions were found to be 70% ethanol, with a liquid to solid ratio of 11:1 (*v*/*w*), a temperature of 70 °C, and a time of 120 min, which gave a yield of polyphenols up to 16.37%. The two major polyphenol compounds were identified to be epicatechin and syringic acid using LC-TOF-MS. The DPPH and reducing power assays showed that ethanol extract of areca seed possessed great antioxidant activity. It is therefore suggested that areca seed is an excellent food material with potential nutritional and antioxidant properties.

The present study suggests that areca seed extracts are useful nutritional antioxidants for the nutraceutical industry with remarkable benefits to human or animal health. However, the key compounds, expected to have been identified by HPLC-MS here, are yet to be determined. Therefore, further study is required to isolate and identify the active antioxidant polyphenol compounds from ethanol extracts of areca seed.
